# Knowledge and Attitudes towards Medical Emergencies among Dentists in Iași, Romania

**DOI:** 10.3390/dj12060148

**Published:** 2024-05-21

**Authors:** Alice Murariu, Livia Bobu, Simona Stoleriu, Roxana-Ionela Vasluianu, Gianina Iovan, Gabriela Luminița Gelețu, Vasilica Toma, Elena-Raluca Baciu

**Affiliations:** 1 Department of Surgicals, Faculty of Dental Medicine, “Grigore T. Popa” University of Medicine and Pharmacy, 700115 Iasi, Romania; alice.murariu@umfiasi.ro (A.M.); livia.bobu@umfiasi.ro (L.B.); gabriela.geletu@umfiasi.ro (G.L.G.); vasilica.toma@umfiasi.ro (V.T.); 2 Department of Cariology, Faculty of Dental Medicine, “Grigore T. Popa” University of Medicine and Pharmacy, 700115 Iasi, Romania; gianina.iovan@umfiasi.ro; 3 Department of Implantology, Removable Prostheses, Dental Technology, Faculty of Dental Medicine, “Grigore T. Popa” University of Medicine and Pharmacy, 700115 Iasi, Romania; elena.baciu@umfiasi.ro

**Keywords:** medical emergencies, knowledge, attitudes, dentists

## Abstract

The aim of this study was to evaluate the level of knowledge of resident dentists and new graduates regarding the etiology, clinical diagnosis, and treatment of the main medical emergency conditions. The study included a sample of 152 new graduates and residents in the first, second, and third year of training in Prosthodontics and General Dentistry from the Faculty of Dental Medicine in Iasi, Romania. Their level of knowledge and the attitudes were assessed using a questionnaire with 24 questions, divided into four sections. The differences among groups were identified using the chi-square test (*p* < 0.05). A high level of knowledge was found among the three groups of subjects for the questions regarding the recognition of clinical signs in hypoglycemic crisis (88–100%), in anaphylactic shock (83.3–94.5%), and the treatment of angina pectoris (76.2–84.2%). In contrast, a low level of knowledge was found for the questions regarding pulse evaluation in the case of an emergency (26.3–35.7%), the parameters of normal breathing (28.9–43%), and the treatment of hypoglycemic crisis (27.8–44.8%). The study indicated that the dentists had a moderate understanding of dental office medical emergencies and preferred practical training over theoretical courses.

## 1. Introduction

A medical emergency represents an acute, unexpected medical phenomenon that endangers the health or life of a patient, necessitating immediate intervention. Dentists can encounter various medical emergencies during dental treatments, ranging from minor issues, such as allergic reactions, characterized by redness and itching, to serious, life-threatening conditions such as anaphylactic shock or myocardial infarction [1,2]. 

Medical emergencies may have various causes. First, the patient’s general diseases, which can sometimes be undiagnosed, may be considered as a risk factor [3]. In addition, elderly patients represent a risk factor for medical emergencies. Poly-pathology and complex drug treatments will increase the risk of drug interactions and the occurrence of adverse effects [4]. In addition, the stress and negative emotions induced by dental treatments in certain categories of patients or long and invasive procedures, such as the surgical ones, also represent risk factors for the occurrence of vasovagal syndrome [5].

The literature indicates that the most frequent medical emergencies encountered in the dental office are syncope, allergic reaction, and hypoglycemia [3]. Cardiac arrest is a very rare emergency in the dental office; nevertheless, dentists must be able to manage this situation, to assess the vital signs, and to perform cardiopulmonary resuscitation maneuvers [6].

Regarding the frequency of occurrence, Muller et al. [7] reported that 57% of the dentists in Germany face three medical emergencies in a 12-month period, and 36% of them face up to 10 medical emergencies. In Saudi Arabia, Alhamad et al. [8] reported a prevalence of 67% of medical emergencies in dental offices over a period of 3 years, and in Slovenia, the prevalence was 67.5% per 12 months [9]. In a review on this topic, Vaughan reported that 43.6% to 75% of dentists must manage a form of medical emergency during their career [10].

Although many studies in the literature investigated the knowledge of dentists regarding the management of emergencies in the dental office, little research on the topic exists in Romania, and the existing studies enrolled only students in the evaluation [11,12].

In a study conducted by Bodnar et al. on 200 patients who applied for specialized treatments in the clinics of the University of Medicine and Pharmacy in Bucharest, the prevalence of medical emergencies was 9%; the most frequently encountered was syncope (27% of the cases), and the most affected age group was 20–40 years [11]. In Brazil, Arsati concluded that anaphylaxis, myocardial infarction, and cardiac arrest were the rarest emergencies, reported by only 0.4, 0.2, and 0.2% of the dentists, respectively [13].

The prevention of medical emergencies that occur during dental treatments is crucial. This can be accomplished by assessing the full medical history of all patients, but especially of those considered to be part of the previously mentioned risk category [4]. The variety of patients who apply for specialized dental treatment is very large. Age, gender, profession, the general pathology are only some of the variables [14]. The medical history of the patient should draw the dentist’s attention to the possibility of medical emergencies to arise [4].

Undoubtedly, it is extremely important for dentists to have solid knowledge about the management of such an episode, to know the methods of providing first aid, and to regularly participate in training courses [15].

To the best of our knowledge, no study has been carried out to assess the knowledge possessed by graduates and resident dentists in Iasi regarding medical emergencies in the dental office.

Therefore, the aim of this study was to investigate the level of knowledge of graduates of the Faculty of Dental Medicine and residents in the specialties of Prosthodontics and General Dentistry regarding medical emergencies in the dental office, starting from the null hypothesis that no differences exist among the three groups of subjects.

## 2. Materials and Methods

### 2.1. Research Design

This cross-sectional study was carried out between April 2023 and January 2024 at the Faculty of Dental Medicine in Iasi, on a group of 152 dental graduates and residents in the specialties of General Dentistry and Prosthodontics.

Prior to study initiation, approval (No. 270/11 February 2023) of the Ethics Committee of “Grigore T. Popa” University of Medicine and Pharmacy in Iasi and the informed consent of study participants were obtained. The subjects were assured that the answers were anonymous, confidential, and their participation was voluntary.

### 2.2. The Study Group

A convenience sampling method was used for subjects’ selection. Initially, 180 graduates with 6 months of experience and residents were invited to participate, of whom only 160 responded to the request. Subsequently, 8 questionnaires were eliminated, as they were incomplete, resulting in a final number of 152 participants with full questionnaires and a participation rate of 84.4%.

The inclusion criteria encompassed dental graduate students or residents in the 1st, 2nd, or 3rd year of training in the specialties of General Dentistry and Prosthodontics at the Faculty of Dental Medicine in Iasi who provided informed consent for participation in the study. On the other hand, the exclusion criteria were defined as students or residents outside the specified specialties, as well as those who declined to participate in the study or provided incomplete questionnaires.

### 2.3. The Study Instrument

A questionnaire was constructed after conducting literature research, appropriate for the study’s purpose [16,17]. 

The questions of the questionnaire were adapted according to the official recommendations of the guide developed by specialists in the management of medical emergencies in Romania [18].

The questionnaire was written in Romanian. The initial form of the questionnaire, written in English, was translated into Romanian by two specialists in linguistics and then re-translated into English. Two experienced university members confirmed the validity of the questionnaire. A preliminary study involving 31 participants from the intended group was conducted to evaluate the questionnaire’s convenience and interpretability. Adjustments were made to the questionnaire based on the findings. A second pilot study was conducted with a group of 15 participants (both residents and graduates), with the aim of verifying some aspects concerning the level of understanding of the questions. After a few necessary adjustments, the final form of the questionnaire was created. The final form contained 24 questions that were tested for internal consistency, resulting in a Cronbach’s alpha coefficient value of 0.730. The completion time was 10 min.

The questionnaire contained 4 sections. The first section included general information: age, gender, year of study, and residency.

The second section included 5 questions (Q1–Q5) that referred to the recognition and measurement of vital signs (pulse, blood pressure, and breathing).

The third section contained 8 questions that presented, in the form of practical scenarios, the most frequent medical emergencies that can occur in dental practice: bronchial asthma (Q6), hypoglycemia (Q7), anaphylactic shock (Q8), stroke (Q9), myocardial infarction (Q10), syncope (Q11), hypertension (Q12), and lidocaine toxicity (Q13).

The last section included 11 questions that focused on participants’ knowledge of the causes, symptoms, and treatment of the following medical emergencies: lidocaine allergy (Q14), syncope (Q15, Q16), anaphylactic shock (Q17), asthma attack (Q18), convulsions (Q19), angina pectoris attack (Q20), and hypoglycemic attack (Q21).

The last 3 questions referred to the frequency of emergencies encountered in current practice (Q22), the perception of the participants regarding their knowledge in this field (Q23), and their desire to participate in additional courses on the topic (Q24).

The questionnaire was distributed to the graduates of the Faculty of Dental Medicine with 6 months of practical activity by online invitation and to the resident doctors during their clinical activities.

### 2.4. The Level of Knowledge

The knowledge score was calculated as follows: correct responses were awarded 1 point, and incorrect responses received 0 points, resulting in a maximum possible score of 21 points for the entire questionnaire. The knowledge level was categorized according to the criteria [19] outlined in Table 1.

### 2.5. Data Analysis

The Statistical Package for Social Sciences program (SPSS Inc., Chicago, IL, USA, version 20 for Windows) was used for the statistical analysis. Frequency, percentage, and crosstabulation were used for descriptive data analysis. The differences between groups were identified using the chi-square test, with a cut-off point of statistical significance of 5% (*p* < 0.05).

## 3. Results

### 3.1. Study Participants

A total of 152 dentists participated in the study, distributed as follows: 42 (27.6%) were resident doctors in the specialty of General Dentistry (GD), 38 (25%) were resident doctors in Prosthodontics (PROSTHOD), and 72 (47.4%) were new graduates of the Faculty of Dental Medicine (GDM) in Iasi. The characteristics of the study participants, based on gender and age distribution, are presented in Table 2.

### 3.2. Participants’ Knowledge about the Characteristics of Vital Signs

The answers given by the study participants to the questions in this section are presented in Table 3.

The correct answer to question Q1 (To record a patient’s pulse, which artery is pressed most frequently?), the radial artery, was identified by the three groups of participants in approximately equal percentages (*p* = 0.265). For question Q2, which refers to recording the pulse in emergency situations, the percentage of correct answers was much lower, especially in the PROSTHOD group (*p* = 0.082). The normal vital signs (Q3) were recognized by a much higher percentage of graduates, compared to the residents of Prosthodontics and the residents of General Dentistry (*p* = 0.022). Tachycardia represented the correct answer to question Q4 (Under what conditions can a pulse with values over 100 beats/minute occur?) and was correctly identified by almost equal percentages of subjects in the three groups (*p* = 0.256). The last question in this section (Q5) refers to normal breathing in an adult patient. The answer was correctly reported by a higher percentage of the GDM graduates, compared to GD residents and PROSTHOD residents (*p* =0.175).

### 3.3. Practical Scenarios Regarding Medical Emergencies in the Dental Office

The questions in this section asked the participants to identify emergency conditions, presented in the form of practical scenarios. The answers are presented in Table 4.

The first question in this section (Q6) presents, as a scenario, the occurrence of an asthma attack. The correct answer was correctly identified by 88.8% of the GDM graduates, but by lower percentages of the GD residents and the PROSTHOD residents (*p* = 0.042). The same trend was found in question Q7, where the hypoglycemic crisis was recognized by a higher percentage of participants, respectively, 100% of the PROSTHOD residents, followed by the graduates and the GD residents (*p* = 0.051). The scenario presented by question Q8 refers to anaphylactic shock, identified by the majority of the study participants (*p* = 0.110). Stroke, the correct answer to question Q9, was correctly identified by the highest percentage of the graduates, followed by the PROSTHOD residents and the GD residents (*p* = 0.003). The correct answer to question Q10, which refers to the recognition of a myocardial infarction, was given by the highest percentage of the graduates, followed by the GD residents and the PROSTHOD residents (*p* = 0.001). For question Q11, participants were asked to recognize the signs of syncope. Most of the correct answers were given by the graduates, while the residents answered correctly at a much lower rate (*p* = 0.035). Significant statistical differences among the three groups of participants (*p* = 0.001) were also observed for question Q12, where the correct answer was identified by most of the graduates and PROSTHOD residents, but by only 54.8% of the GD residents. Question Q13 presents the clinical signs in lidocaine toxicity. Only 54.3% of the graduates correctly identified the signs of this medical emergency, followed by 44.7% of the PROSTHOD residents and 23.8% of the GD residents (*p* = 0.002).

### 3.4. Participants’ Knowledge about the Etiology, the Clinical Signs, and the Treatment of the Main Emergencies in the Dental Office

The answers given to the questions in Section 4 are presented in Table 5.

**Figure 1:**
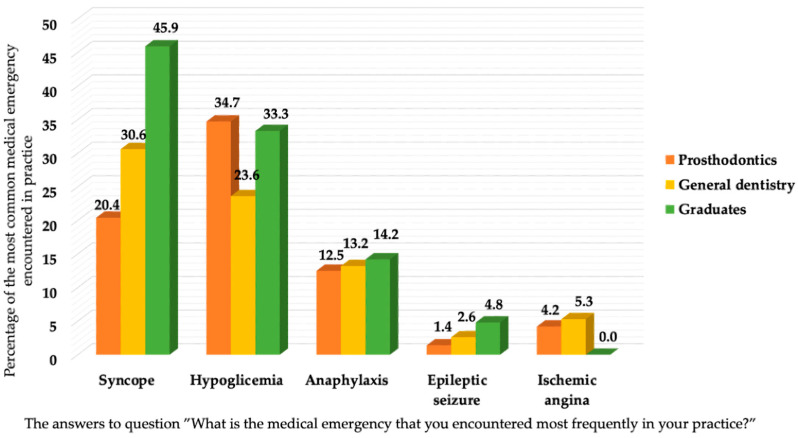
The answers to question Q22: What is the medical emergency that you encountered most frequently in your practice?

The differences observed in the percentages of correct answers to question Q14 were statistically significant (*p* = 0.005): the percentage of correct answers provided by the graduates was much higher than that of the residents. The following questions refer to the etiology (Q15) and the treatment (Q16) of vasovagal syncope. The correct answer to Q15 was known by the three groups of subjects in almost equal percentages (*p* = 1.020). The correct treatment of syncope (Q16) was correctly identified by only a small percentage of subjects: 60.5% of the PROSTHOD residents, 47.6% of the GD residents, and 48.7% of the graduates (*p* = 0.044). In the case of anaphylactic shock (Q17), the correct treatment consists of the administration of adrenaline; this answer was given by more than 80% of the participants (*p* = 0.235). The correct answer to question Q18 (What is the treatment for an asthma attack?) was given by the highest percentage of the graduates, followed by the GD residents and the PROSTHOD residents (*p* = 0.627). The percentage of correct answers to question Q19 (What is the therapeutic attitude in the case of convulsions?) varied from 50% to 66.6% (*p* = 0.056). Question Q20 refers to the treatment in cases of retrosternal chest pain. The frequency of correct answers was the highest among the PROSTHOD residents (*p* = 0.293). Question Q21 refers to emergency treatment in the event of a hypoglycemic crisis. The correct answer (oral administration of carbohydrates) was known by only a small percentage of the participants (44.8% of the PROSTHOD residents, 38% of the GD residents, and 27.8% of the graduates). In most cases, confusion was observed regarding the treatment option, as the intravenous administration of glucose was selected as the correct answer by a large percentage of the GD residents (50%) and the graduates (50%) (*p* = 0.002). The most frequent medical emergency that was encountered in practice (Q22) was syncope (identified by 45.9% of the graduates), followed by hypoglycemic crisis (identified by 34.7% of the PROSTHOD residents), anaphylaxis (14.2% of the graduates), and very few cases of convulsions (4.8% of the graduates) and ischemic angina (5.3% of the GD residents) (Figure 1).

The levels of knowledge regarding the responses to the questions from the three sections are presented in Table 6.

Regarding satisfaction with the knowledge held in the field of medical emergencies (Q23), the percentage of respondents who answered that they were satisfied with the acquired knowledge varied from 62.5% for the graduates to 45.2% for the GD residents, and 44.7% for the PROSTHOD residents (*p* = 0.009). Almost half of the participants declared that they were willing to receive additional information regarding the management of medical emergencies (Q24) in the form of scenarios or simulations, rather than in the form of theoretical courses (*p* = 0.079).

## 4. Discussion

The statistical analysis led to the rejection of the null hypothesis, thereby confirming the existence of differences in knowledge among the three categories of study participants.

A series of publications on the knowledge and management of medical emergencies in the dental office are cited in the literature [20,21,22,23,24,25,26,27]. Some focus on first aid measures, others focus on the responsibility of dentists and the degree of satisfaction with their own skills and abilities. In the present paper, we aimed to assess the knowledge of young dentists regarding the causes, the clinical signs, and the treatment of various types of emergencies that may occur during dental treatments.

Nevertheless, medical emergencies in the dental office put the patient’s life at risk and they must be treated quickly and with vigilance. Therefore, the doctor must evaluate the patient’s vital signs and must be able to manage any changes. The results of the present study indicate a low level of knowledge regarding the method of pulse evaluation in the case of emergencies (Q2). The artery that must be pressed in this situation is the carotid artery, not the radial artery, which is pressed in normal situations. The frequency of correct answers varied from 26.3% to 35.7%. A percentage of 44.8% to 51.4% of participants incorrectly considered that the radial artery should be pressed in such circumstances. Regarding the number of breaths per minute (Q5), the percentage of respondents who recognized the normal values was reduced: only 28.9% of the PROSTHOD residents and 43% of the graduates provided correct answers. However, when compared to similar studies, the percentage in our study is high. In India, for instance, the percentage of correct answers was 19.75% for graduates and 30.19% for dental practitioners [1]. This circumstance can be attributed to the lack of practical training modules involving mannequins in the residency curriculum for first aid techniques, underscoring the imperative for their use.

The participants of the present study answered that syncope was the most frequent of all medical emergencies, as reported by both graduates and residents with longer practice experience. The literature also indicates a higher frequency of this type of medical emergency: 53.1% in Saudi Arabia [8], 46.3% in Poland [6], and 54.2% in Brazil [13]. Because of its high frequency of occurrence, dental practitioners must be aware of the causes and the therapeutic procedures in the case of syncope [28]. 

The clinical manifestations of syncope were recognized by a higher percentage of the graduates (81.9%), compared to the residents in Prosthetic Dentistry (68.5%) and the residents in General Dentistry (69%). A moderate level of knowledge was found for questions regarding the cause of syncope (65.2–66.6%), as well as the correct positioning of the patient, where the percentage varied from 47.6% for the residents of General Dentistry to 60.5% for the residents of Prosthetic Dentistry. A possible explanation for these responses might be that the participants have limited experience in the field, and they have not encountered many cases in their current practice yet. Fernandez et al. [1] reported similar percentages of respondents (both students and practitioners) with correct answers, while in the study conducted by Ahamed et al. [29], all the participants provided correct answers. In a review of the literature, Hutse et al. [28] reported a percentage of 79.2% of dentists who were able to correctly diagnose syncope, but most of them (86%) did not have the necessary skills for an adequate treatment.

The answers to the questionnaire used in the present study indicated a high level of knowledge of the respondents for certain questions/scenarios, such as the questions regarding the clinical signs and the emergency treatment of anaphylactic shock by adrenaline administration (Q8, Q17). In Chennai, in a similar research study, the authors reported that 68% of the dentists specified the correct treatment for anaphylactic shock [30], a percentage much lower than the ones found in the present study (80.6% for the graduates and 89.5% for the PROSTHOD residents). In contrast, the study carried out in Saudi Arabia by Albelaihi et al. [31] reported that 40% of the participants chose antihistamines as the first choice in the drug treatment of anaphylaxis, and only 33% of them chose adrenaline. Smereka et al. [6] found that 53% of the Polish dentists considered that they were competent in carrying out the treatment for minor allergic reactions, and 42% in the treatment of anaphylactic shock. 

Additionally, a high level of knowledge was found concerning the medical management of retrosternal chest pain through the sublingual administration of nitroglycerin (Q20), as well as the recognition of the clinical signs of hypoglycemia (Q7). This level of knowledge was suggested by the percentage of correct answers, varying from 88% to 100% in the case of hypoglycemia and from 76.2% to 83.3% in the case of nitroglycerin administration. In previous research on this topic, Nagarale et al. [32] found a percentage of 86.66% of correct answers when referring to hypoglycemic crisis and 94.5% when referring to the treatment of retrosternal pain with nitroglycerin.

On the other hand, for four questions in this section, a low level of knowledge was found across all categories of participants. These include question Q21, concerning the therapeutic approach in hypoglycemic crisis, where a large percentage of practitioners (50% of the GD residents, 50% of the graduates, and 39.4% of the PROSTHOD residents) considered that the intravenous administration of glucose was the correct intervention, instead of the oral administration of carbohydrates. A similar situation was found for question Q19, concerning the therapeutic intervention in the case of convulsions, where many participants considered that the intravenous administration of diazepam was the treatment of choice.

Local and regional anesthesia are widely used in dental therapy. This means that specialists must correctly identify the clinical signs of lidocaine toxicity (Q13). The level of knowledge for this question was moderate for the graduates and low for the residents, who considered the signs as being specific for an allergic reaction (35.8% of the GD residents) or a hypoglycemic crisis (10.5% of the PROSTHOD residents). Almost one third of the respondents did not provide the correct answer to this question. Another question regarding dental anesthesia was Q14, where the etiologic aspect of an allergic reaction during the anesthetic puncture was emphasized. The percentage of correct answers was over 50% for all the participants, but incorrect answers were also found, such as those given by 21% of the PROSTHOD residents and 19.1% of the GD residents. 

Differences in the level of knowledge among the three groups of respondents were observed in the present study. Specifically, 43.1% of the graduates were able to correctly answer 16 questions or more, while a smaller percentage of the residents in Prosthodontics and General Dentistry achieved this. The possible explanation of the observed difference between the participants would be that during their studies, the discipline of Medical Emergencies is provided in the academic curriculum in the 6th year of study, meaning that the graduates have knowledge that was recently taught. Regarding the residency curriculum for the two specialties, there are no courses/seminars on this topic, and young dentists have only the knowledge and skills acquired during university, which sometimes seem insufficient.

All the respondents in the present study acknowledged the need for additional courses in this field. The subject of medical emergencies should be introduced in the residency curriculum, and postgraduate education should regularly include courses/seminars/exercises and practical scenarios. In addition, dental practitioners must be familiar with the appropriate drugs and equipment necessary for managing medical emergencies [33].

Whenever such a situation occurs, the doctor must act quickly, must have the necessary knowledge and skills, rely on them, and remain calm during the management of the medical emergency [34]. In Romania, according to the College of Dentists, it is mandatory for every dental practice to have an emergency medical kit that includes equipment, materials, and medicines to cover the most important medical emergencies [35]. Six medications should be considered essential: oxygen, epinephrine, nitroglycerin, antihistamines, albuterol/salbutamol, and aspirin. Beside these, others are very useful and should be included in the emergency kit: glucagon, atropine, ephedrine, hydrocortisone, morphine or nitrous oxide, lorazepam or midazolam, and flumazenil [36].

This study has some limitations. First, the questionnaire did not include questions about first aid maneuvers, but only about the knowledge of vital signs. Second, the internal consistency of the questionnaire used was not very high [37]. However, the analysis of the relationship between each item and the total scale did not indicate a significant increase in the Cronbach’s alpha coefficient by eliminating any of the items, which is why it was preferred to keep them, given the relevance of each in evaluating the level of knowledge. The third limitation is that the study included only resident doctors from two specializations; therefore, its results cannot be considered representative of the knowledge of all categories of resident doctors. In the future, this kind of research can be extended to include dentists more experienced in dental practice. 

## 5. Conclusions

Following this study, we can conclude that the level of knowledge of the participants in the study is moderate, as we found that the highest number of correct answers fell within the 51–75% level. A comparative analysis between the three categories of participants showed that the most correct answers were provided by the graduates, followed by the residents of Prosthetic Dentistry and the residents of General Dentistry. Another finding is that all participants have modest knowledge regarding the correct recording of vital signs, pulse, and respiration.

This study highlighted the need to add the subject of medical emergencies to the university curriculum for both students and resident doctors, as well as to update/introduce professional practical guides. Dental associations should play an important role in increasing the practitioners’ level of knowledge by organizing continuous medical training courses periodically.

## Figures and Tables

**Table 1:** Level classification of total knowledge scores.

Knowledge Scores	Percent	Knowledge Level
1–10	less than 50%	Low
11–15	51–75%	Moderate
16–21	76–100%	Good

**Table 2:** Characteristics of study participants (N = 152).

Variables		Total N (%)	Graduates of Dental Medicine Faculty N (%)	General Dentistry Residents N (%)	Prosthodontics Residents N (%)
Gender	Male	61 (40.13)	26 (42.6)	24 (39.3)	11 (18)
	Female	91 (59.87)	46 (50.5)	18 (19.8)	27 (29.7)
Total N (%)		152 (100)	72 (47.4)	42 (27.6)	38 (25)
Age (mean ± SD ^1^) 27.01 ± 0.350	25–30 years	123 (80.9)	58 (47.1)	38 (30.9)	27 (22)
	31–35 years	17 (11.2)	10 (58.8)	2 (11.8)	5 (29.4)
	36–40 years	12 (7.9)	4 (33.3)	2 (16.7)	6 (50)
Total N (%)		152 (100)	72 (47.4)	42 (27.6)	38 (25)

^1^ SD: standard deviation.

**Table 3:** Participants’ answers to the questions in Section 2—Knowledge of vital signs. Correct answers are marked in italics.

	Prosthodontics Residents (N = 38)	General Dentistry Residents (N = 42)	Graduates (N = 72)	*p*-Value
	N (%)	N (%)	N (%)	
Q1. To record a patient’s pulse, which artery is pressed most frequently?				
Brachial artery	2 (5.2)	0	6 (8.3)	0.265
Carotid artery	5 (13.2)	4 (9.6)	8 (11.1)	
*Radial artery*	28 (73.7)	31 (73.8)	52 (72.3)	
I don’t know	3 (7.9)	7 (16.6)	6 (8.3)	
Q2. Which artery is pressed in the case of a patient with medical emergencies?				
Brachial artery	2 (5.2)	0	5 (6.9)	0.082
*Carotid artery*	10 (26.3)	15 (35.7)	25 (34.8)	
Radial artery	17 (44.8)	21 (50)	37 (51.4)	
I don’t know	9 (23.7)	6 (14.3)	5 (6.9)	
Q3. Which one of the following scenarios most accurately illustrates normal vital signs?				
Pulse 90, BP 140/120	15 (39.4)	13 (30.9)	9 (12.5)	0.022 *
*Pulse 60–80, BP 120/70*	14 (36.8)	20 (47.6)	59 (82)	
Pulse 70, BP 100/80	5 (13.2)	5 (11.9)	4 (5.5)	
I don’t know	4 (10.6)	4 (9.6)	0	
Q4. Under what conditions can a pulse with values over 100 beats/minute be found?				
Arrythmia	6 (15.8)	4 (9.6)	10 (13.8)	0.256
*Tachycardia*	28 (73.7)	31 (73.8)	57 (79.3)	
Hypertension	3 (7.8)	5 (11.9)	5 (6.9)	
I don’t know	1 (2.7)	2 (4.7)	0	
Q5. What is the normal breath/minute for adult patients?				
20 breaths per minute	13 (34.2)	7 (16.6)	10 (13.8)	0.175
*12–18 breaths per minute*	11 (28.9)	16 (38)	31 (43)	
Over 25 breaths per minute	4 (10.6)	10 (23.9)	14 (19.5)	
I don’t know	10 (26.3)	9 (21.5)	17 (23.7)	

Notes: BP = blood pressure; * significance level of 0.05 (Chi square test).

**Table 4:** Participants’ answers to the questions in Section 3—Practical scenarios. Correct answers are marked in italics.

	Prosthodontics Residents	General Dentistry Residents	Graduates	*p*-Value
	N = 38	N = 42	N = 72	
	N (%)	N (%)	N (%)	
Q6. In the case of a patient with dyspnea, wheezing, sweating, perioral cyanosis, what do you suspect?				
*Bronchial asthma*	26 (68.4)	27 (64.2)	64 (88.9)	0.042 *
Anaphylactic shock	8 (21)	4 (9.6)	4 (5.6)	
Myocardial infarction	4 (10.6)	6 (14.3)	1 (1.4)	
I do not know	0	5 (11.9)	3 (4.1)	
Q7. In the case of a diabetic patient with sweating, tremors, disorientation, headache, what do you suspect?				
*Hypoglycemic crisis*	38 (100)	37 (88.1)	69 (95.9)	0.051
Anaphylactic reaction	0	0	0	
Angina pectoris	0	0	0	
I do not know	0	5 (11.9)	3 (4.1)	
Q8. In the case of a patient who suddenly develops swelling of the face and lips, dyspnea, what do you suspect?				
*Anaphylactic shock*	34 (89.5)	35 (83.3)	68 (94.5)	0.110
Transient ischemia	0	0	0	
Angina pectoris	1 (2.7)	1 (2.4)	3 (4.1)	
I do not know	3 (7.8)	6 (14.3)	1 (1.4)	
Q9. In the case of an elderly patient who shows restlessness, slurred speech, hypersalivation during treatment, what do you suspect?				
*Stroke*	33 (86.9)	31 (73.8)	68 (94.5)	0.003 *
Myocardial infarction	1 (2.6)	0	3 (4.1)	
Hypoglycemic crisis	3 (7.9)	7 (16.6)	0	
I do not know	1 (2.6)	4 (9.6)	1 (1.4)	
Q10. In the case of a patient with dizziness, sweating, retrosternal pain, pain in the left jaw, what do you suspect?				
*Myocardial infarction*	26 (68.4)	29 (69)	71 (98.6)	0.001 *
Bronchial asthma	0	1 (2.4)	0	
Syncope	9 (23.7)	12 (28.6)	1 (1.4)	
I do not know	3 (7.9)	0	0	
Q11. In the case of a pale patient with sweats, pallor, cold extremities, weak pulse, dizziness, loss of consciousness, what do you suspect?				
*Syncope*	26 (68.5)	29 (69)	59 (81.9)	0.035 *
Myocardial infarction	4 (10.5)	6 (14.3)	10 (13.9)	
Hypoglycemic crisis	8 (21)	5 (11.9)	2 (2.8)	
I do not know	0	2 (4.8)	1 (1.4)	
Q12. In the case of a patient with agitation, dyspnea, dizziness, strong occipital and frontal headache, what do you suspect?				
*High blood pressure crisis*	36 (94.7)	23 (54.8)	71 (98.6)	0.001 *
Angina pectoris	0	16 (38)	1 (1.4)	
Anaphylactic shock	2 (5.3)	1 (2.4)	0	
I do not know	0	2 (4.8)	0	
Q13. During the administration of local anesthesia to a patient who becomes anxious, has tremors, tinnitus, blurred vision, what do you suspect?				
*Lidocaine toxicity*	17 (44.7)	10 (23.8)	39 (54.3)	0.002 *
Hypoglycemic crisis	4 (10.5)	3 (7.2)	7 (9.7)	
Allergic reaction	6 (15.8)	15 (35.6)	7 (9.7)	
I do not know	11 (29)	14 (33.4)	19 (26.3)	

Note: * significance level of 0.05 (chi-square test).

**Table 5:** Participants’ answers to the questions in Section 4—Knowledge regarding medical emergencies, the treatment provided, and continuing medical education. Correct answers are marked in italics.

	Prosthodontics Residents N = 38	General Dentistry Residents N = 42	Graduates N = 72	*p*-Value
	N (%)	N (%)	N (%)	
Q14. When can an allergic reaction occur during local anesthesia?				
*During or after the administration of the anesthetic dose*	27 (71)	28 (66.7)	68 (94.5)	0.005 *
Incorrect administration	2 (5.3)	5 (11.9)	4 (5.5)	
Fast administration	8 (21)	8 (19.1)	0	
I do not know	1 (2.7)	1 (2.3)	0	
Q15. What is the main cause of syncope in the dental office?				
*Stress and anxiety*	25 (65.7)	28 (66.6)	47 (65.2)	1.020
Hunger	6 (15.8)	6 (14.3)	7 (9.8)	
Prolonged treatment	3 (7.9)	5 (11.9)	13 (18.1)	
I do not know	4 (10.6)	3 (7.2)	5 (6.9)	
Q16. What should you do in case of a syncope in the dental office?				
*Placing the patient in a supine position with the head lower than the body*	23 (60.5)	20 (47.6)	35 (48.7)	0.044 *
Placing the patient in lateral decubitus	6 (15.7)	14 (33.4)	11 (15.2)	
Placing the patient in the prone position	5 (13.2)	2 (4.7)	6 (8.3)	
I do not know	4 (10.6)	6 (14.3)	20 (27.8)	
Q17. What is the elective treatment in case of an anaphylactic shock?				
*Adrenalin*	34 (89.4)	34 (81)	58 (80.6)	0.235
Antihistamines	0	2 (4.7)	0	
Cortisone	4 (10.6)	4 (9.6)	10 (13.9)	
I do not know	0	2 (4.7)	4 (5.5)	
Q18. What is the treatment of choice in case of an asthma attack?				
*Bronchodilator*	25 (65.7)	28 (66.7)	55 (76.3)	0.627
Cortisone	13 (34.3)	13 (30.9)	16 (22.3)	
I do not know	0	1 (2.4)	1 (1.4)	
Q19. What is the therapeutic attitude in cases of convulsions?				
*Placing the patient in lateral decubitus and emptying the oral cavity*	25 (65.7)	21 (50)	48 (66.7)	0.056
Placing the patient in the supine position and emptying the oral cavity	0	1 (2.4)	2 (2.8)	
Administration of Diazepam iv	13 (34.3)	20 (47.6)	17 (23.6)	
Placing the patient in a vertical position	0	0	4 (5.5)	
I do not know	0	0	1 (1.4)	
Q20. What is administered to a patient with retrosternal pain?				
*Sublingual nitroglycerin*	32 (84.2)	32 (76.2)	60 (83.4)	0.293
Physiological serum iv	1 (2.6)	1 (2.4)	1 (1.4)	
Nifedipine	4 (10.6)	8 (19)	5 (6.9)	
I do not know	1 (2.6)	1 (2.4)	6 (8.3)	
Q21. What is the therapeutic attitude in the event of a hypoglycemic crisis?				
*Administration of carbohydrates orally*	17 (44.7)	16 (38.1)	20 (27.8)	0.002 *
Administration of glucose iv	15 (39.4)	21 (50)	36 (50)	
Physiological serum iv	4 (10.6)	2 (4.7)	10 (13.9)	
I do not know	2 (5.3)	3 (7.2)	6 (8.3)	
Q23. Are you satisfied with the knowledge you have in the field of medical emergencies?				
Yes	17 (44.7)	19 (45.2)	45 (62.5)	0.009 *
No	21 (55.3)	23 (54.8)	27 (37.5)	
Q24. In what form would you like to get additional information?				
I do not want additional information	0	1 (2.4)	2 (2.8)	0.079
Practical scenarios/simulation/workshop	20 (52.6)	24 (57.1)	38 (52.7)	
Courses	4 (10.6)	8 (19)	22 (30.6)	
Exercises	14 (36.8)	9 (21.5)	10 (13.9)	

Note: * significance level of 0.05 (chi-square test).

**Table 6:** Levels of knowledge.

Variables	Knowledge Level			*p*-Value
	Low N (%)	Moderate N (%)	Good N (%)	
Graduates	1 (1.4)	40 (55.6)	31 (43.1)	0.00 *
General Dentistry Residents	2 (4.8)	37 (88.1)	3 (7.1)	
Prosthodontics Residents	1 (2.6)	32 (84.2)	5 (13.2)	

Note: * significance level of 0.05 (chi-square test).

## Data Availability

The data that support the findings of this study are available on request from the corresponding author.
